# Cellular factories for coenzyme Q_10_ production

**DOI:** 10.1186/s12934-017-0646-4

**Published:** 2017-03-02

**Authors:** Sean Qiu En Lee, Tsu Soo Tan, Makoto Kawamukai, Ee Sin Chen

**Affiliations:** 1 Department of Biochemistry, National University of Singapore, Singapore, Singapore; 2 National University Health System (NUHS), Singapore, Singapore; 3 NUS Synthetic Biology for Clinical and Technological Innovation (SynCTI), Life Sciences Institute, National University of Singapore, Singapore, Singapore; 4 NUS Graduate School for Integrative Sciences and Engineering, National University of Singapore, Singapore, Singapore; 5 School of Chemical & Life Sciences, Nanyang Polytechnic, Singapore, Singapore; 6 Faculty of Life and Environmental Science, Shimane University, Matsue, 690-8504 Japan

**Keywords:** Coenzyme Q_10_, Isoprenoid, Antioxidant, Industrial biosynthesis, Protein engineering, Synthetic biology

## Abstract

Coenzyme Q_10_ (CoQ_10_), a benzoquinone present in most organisms, plays an important role in the electron-transport chain, and its deficiency is associated with various neuropathies and muscular disorders. CoQ_10_ is the only lipid-soluble antioxidant found in humans, and for this, it is gaining popularity in the cosmetic and healthcare industries. To meet the growing demand for CoQ_10_, there has been considerable interest in ways to enhance its production, the most effective of which remains microbial fermentation. Previous attempts to increase CoQ_10_ production to an industrial scale have thus far conformed to the strategies used in typical metabolic engineering endeavors. However, the emergence of new tools in the expanding field of synthetic biology has provided a suite of possibilities that extend beyond the traditional modes of metabolic engineering. In this review, we cover the various strategies currently undertaken to upscale CoQ_10_ production, and discuss some of the potential novel areas for future research.

## Background

**Fig. 1:**
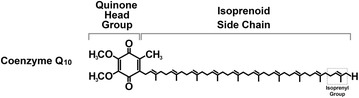
Chemical structure of coenzyme Q_10_. This molecule consists of a isoprenoid side chain composed of ten tandemly linked isoprenyl groups attached to a quinone head group

Coenzyme Q, commonly known as ubiquinone or CoQ, is a lipid-soluble, powerful antioxidant, and an essential cofactor in mitochondrial oxidative phosphorylation [1–3]. Coenzyme Q is species specific, with differences dictated by the number of isoprenyl units on the isoprenoid side chain. For example, 10 isoprenyl units are found in human and the fission yeast *Schizosaccharomyces pombe* but fewer units are found in other species (CoQ_8_ in *Escherichia coli,* CoQ_9_ in *Arabidopsis thaliana*, and CoQ_6_ in *Saccharomyces cerevisiae*) [1]. The isoprenoid side chain is responsible for the lipid-soluble nature of CoQ, whereas its antioxidant capacity derives from its quinone head, which can enable electron transfer (Fig. 1). Because of this electron-sequestering property, CoQ_10_ acts as an antioxidant at cellular membranes to counteract the oxidation of lipids or lipoproteins [4]. CoQ_10_ has roles in other physiological processes, including sulfide oxidation, regulating the mitochondrial permeability transition pore, and in the translocation of protons and Ca^2+^ across biological membranes [5, 6]. A detailed account of the various aspects of CoQ biosynthesis have been described at length elsewhere [1–6].

CoQ_10_ is the only lipid-soluble antioxidant produced by humans, and it localizes to almost every membrane, ranging from mitochondrial membranes to that of very low density lipoproteins (VLDL) [7]. This solubility means that CoQ can protect lipoproteins and lipids from peroxidation and oxidative damage [8]. CoQ_10_ also serves alongside other antioxidants, such as vitamins C and E, to combat free-radical damage arising from energetic mitochondrial reactions [9, 10]. Given its myriad functions and physiological importance, it is not surprising that CoQ deficiency can result in numerous diseases.

In model organisms, such as *S. cerevisiae* and *S. pombe*, CoQ deficiency is not lethal but results in growth defects on minimum medium, and a heightened sensitivity to oxidative stress [11–15]. In *Caenorhabditis elegans*, CoQ deficiency leads to GABA neuron degeneration, and in *Drosophila melanogaster*, it can cause mitochondrial stress and neuronal apoptosis [16, 17]. In humans, CoQ_10_ deficiency has been implicated in various diseases involving muscle and neural development, with the severity of the disease correlated with the acuteness of the CoQ_10_ shortfall [18]. These diseases may manifest in conditions such as central nervous system (CNS) dysfunction, myopathy or cardiomyopathy, among others [19–22]. The oxidative damage associated with impaired CoQ_10_ function has also been implicated in numerous clinical phenotypes [18, 23–25].

By virtue of its therapeutic relevance, CoQ_10_ is of particular importance in the biomedical and health supplement scene. Oral CoQ_10_ supplements are often prescribed alongside treatments for various diseases [26]. One example is its co-administration with HMG-CoA (3-hydroxy-3-methylglutaryl-coenzyme A) reductase (HMGR) inhibitors, widely used cholesterol-lowering drugs otherwise known as statins. HMGR catalyzes the formation of mevalonic acid, the precursor for cholesterol and CoQ_10_ biosyntheses [27]. Patients using statins show lower blood levels of CoQ_10_, and this justifies the need for CoQ_10_ supplementation to reduce the cardiomyopathy risk associated with statin use [27–30]. The presence of CoQ_10_ is however implicated in resistance to chemotherapeutic drugs, and this calls for caution in administering CoQ_10_ alongside certain agents [31, 32].

CoQ_10_ production decreases with aging [33], as does the antioxidant capability of the cell. Increased oxidative stress in aging cells may be ameliorated with dietary supplementation of CoQ_10_ [34]. Indeed, CoQ_10_ has garnered great popularity as an antioxidant in moisturizers, anti-wrinkle and anti-aging skin care treatments [35–37]. With the growing demand for skin care cosmetics and public awareness of the importance of antioxidants, we will likely see an increase in the demand for CoQ_10_ products on the market quite quickly [38]. Given that CoQ_10_ is endogenously synthesized, there should be fewer unwanted side effects from its therapeutic use as compared with other synthetic compounds, and this has been supported by tolerability studies for high CoQ_10_ doses [39]. Hence, attention has surged in the therapeutic use of CoQ_10_ in non-curable diseases challenging modern societies including Alzheimer’s, Huntington’s and Parkinson’s, and cardiovascular diseases [40–42].

### Industrial production of CoQ_10_

The range of uses for CoQ_10_ across the pharmaceutical and cosmetics industries has meant that there is great commercial interest to scale up the production of CoQ_10_. Frederick Crane first isolated CoQ_10_ from a bovine heart source in the late 1950s [43]. Since then, industrial attempts to produce CoQ_10_ have centered on animal tissue extraction, semi-chemical synthesis, and microbial fermentation [44, 45].

The chemical synthesis of CoQ_10_ has typically involved solanesol as a starting substrate and the source of the isoprenoid tail, and this is carried out before it is combined with the quinone head [46]. However, as with most chemical processes, there are numerous costs associated with such high-energy catalysis reactions because of the need for expensive substrates and because of the significant chemical waste generated from its production [47–49]. The chemical synthesis of CoQ_10_ also lacks stereoselectivity, and this makes it difficult to separate optical isomers to obtain the all-*trans* biologically viable isomer [50].

Owing to these difficulties, microbial biosynthesis has become a preferred avenue of CoQ_10_ production. The cell-based catalysis of compounds does not require harsh catalytic conditions of heat and pressure that typify many chemical synthesis processes. Furthermore, the production costs tend to be lower, cheap growth media provides an appropriate substrate, and expensive co-substrates can be recycled [48, 51]. A living cellular system is also scalable, and the precision of the cellular catalytic machinery circumvents the problems of stereoselectivity [52, 53]. Furthermore, unlike with chemical processing, altered genetics does not significantly affect the operating costs, meaning that the efforts associated with constructing a high-titer-producing organism are worthwhile. Through microbial biosynthesis, metabolic engineering approaches can be used to increase the titer of CoQ_10_ and overcome some of the limiting steps along the biosynthetic pathway.

Metabolic engineering approaches initially used chemical mutagenesis-based selection and chemical engineering procedures that centered on manipulating substrate flux; however, the field has since expanded to include other strategies from a genetics standpoint [48, 54]. The process varies depending on promoter choice and strength, cassette copy number, and the localization or tethering of enzymes to scaffolds [55, 56]. The choice of cassette and promoter are typically host dependent, given that promoter strength and usability rely on a species-specific genetic environment and functionality. Furthermore, enzymes involved in the tail end of CoQ_10_ production are localized in the mitochondria, leading to models that propose the involvement of a membrane-bound complex containing multiple polypeptides of the CoQ_10_ biosynthesis enzymes [57].

Improving flux remains one of the most straightforward methods to increase yield [48, 58]. Typically, this involves finding and circumventing rate-limiting steps in biochemical pathways and then employing strong promoters to increase the expression of key pathway genes to direct biochemical flux. A parallel option entails knocking down the expression of genes in alternate pathways that branch off the pathway of interest, and this can be concomitantly administered, with care taken to ensure that these manipulations do not undermine cellular viability and robustness. Alleviating chemical bottlenecks that might hamper the production of the desired compound can also be achieved by including genes that reconstitute cofactors, such as NADPH and S-adenosyl methionine (SAM). These cofactors play essential roles in numerous biochemical pathways [54, 59]. Overall, it is clear that close scrutiny and careful optimization of biosynthetic pathways can optimize and direct the metabolic flux.

### Biosynthesis of CoQ_10_

#### Entry points to CoQ_10_ biosynthesis

**Fig. 2:**
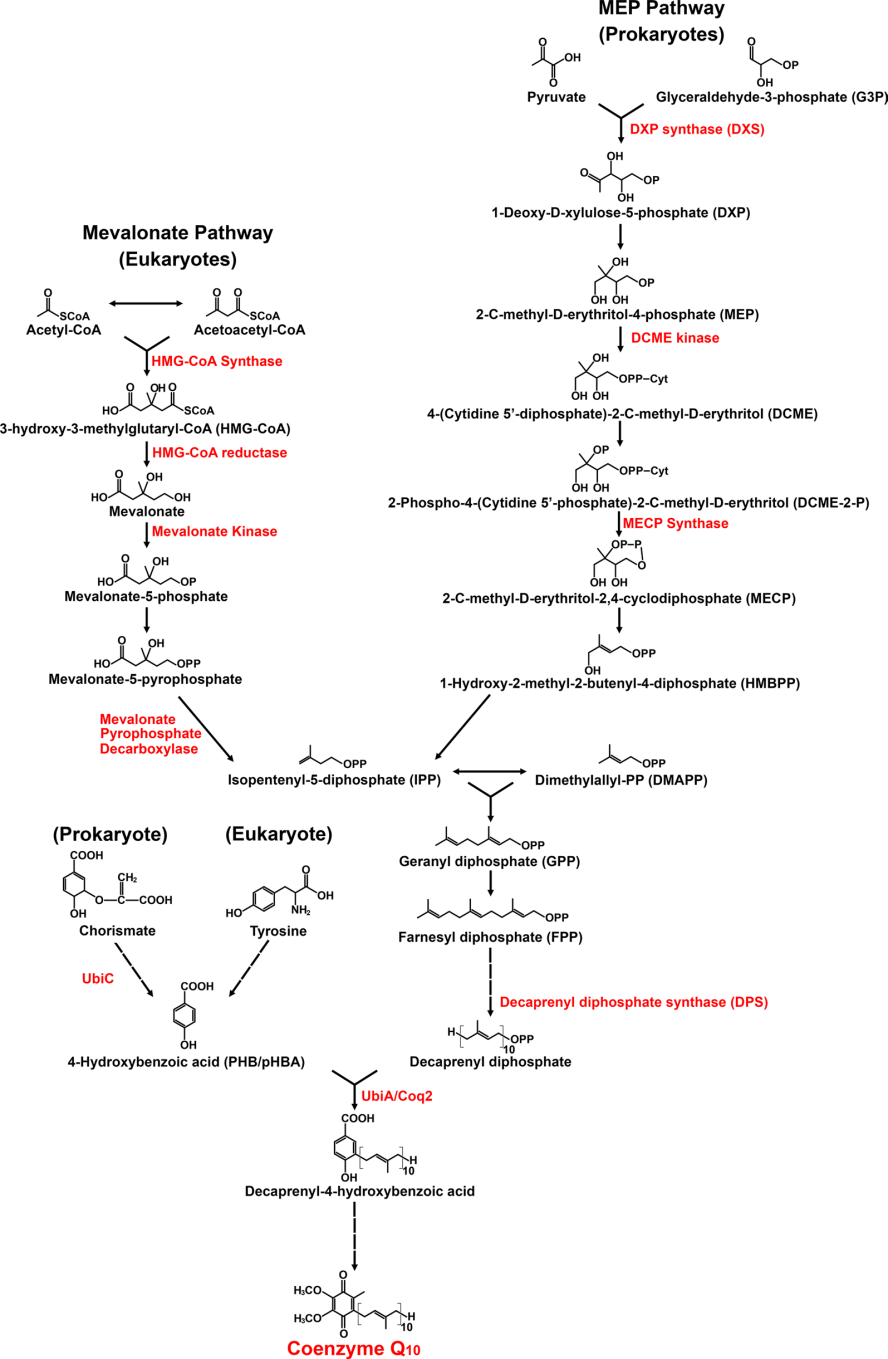
Biosynthesis of coenzyme Q_10_. Schematic showing the pathway of various metabolic precursors leading to the formation of the quinone head (PHB), the isoprene tail (decaprenyl diphosphate), and the final Coenzyme Q product. Reflected in *red* are the various enzymatic steps that are rate limiting. UbiC and UbiA are specific genes from *E. coli*, and Coq2 is from *S. cerevisiae*. Unlabelled *arrows* between chorismate and tyrosine and PHB; FPP and decaprenyl diphosphate; and decaprenyl-4-hydrobenzoic acid and coenzyme Q_10_ denote the presence of multiple steps that have been abbreviated

CoQ biosynthesis involves discrete synthetic stages: production of the aromatic group that forms the quinone head, production of the isoprene tail, attachment of the quinone head to the isoprene tail, and the subsequent steps that culminate in the formation of the final CoQ_10_ product [1, 60]. In yeast, mitochondria are responsible for CoQ synthesis. However, in humans, both mitochondria and Golgi apparatus are proposed sites for CoQ synthesis. The chemical precursors for both the quinone head and isoprene tail are organism specific. The quinone head is derived from the chorismate precursor in the shikimate pathway in prokaryotes but from tyrosine in higher eukaryotes (Fig. 2). The isoprene tail derives from MEP (2-C-methyl-d-erythritol 4-phosphate) in prokaryotes and plant plastids, which stems from glyceraldehyde 3-phosphate (G3P), whereas, in eukaryotes, the tail is produced from acetyl-CoA in the mevalonate pathway [2, 61]. These multiple entry points into the pathway could be exploited to optimize flux for yield improvement.

The engineering concept of ‘push and pull’ to divert metabolic flux implicates that both the inflow and outflow reactions must be increased synchronously, otherwise an accumulation of one product will limit the flux and cause an imbalance in the system. Therefore, it is crucial to understand the species-specific biosynthetic pathways that lead to CoQ_10_ production. There are several biosynthetic pathways of concern, each of which we will address separately.

#### Rate-limiting steps in biosynthesis of the isoprenoid chain

The first pathway provides the precursors for synthesizing the isoprene tail; if using a prokaryotic system, this is achieved through the MEP pathway. The MEP pathway starts with the interaction between G3P and pyruvate to form 1-deoxy-d-xylulose 5-phosphate (DXP) (Fig. 2), which is reported to be the major limiting step in the formation of the isoprene tail [62]. Indeed, efforts to increase the prokaryotic expression of carotenoids (which share the isoprenoid precursor pathway of MEP) have focused on improving the first catalytic step of DXP formation. Under such contexts, 1-deoxy-d-xylulose-5-phosphate synthase (DXS) and 1-deoxy-d-xylulose 5-phosphate reductoisomerase (DXR) are typically overexpressed to improve the catalytic formation of DXP and its subsequent conversion to MEP [60]. These reactions eventually yield isopentenyl diphosphate (IPP), which is used to initiate isoprene chain elongation in the isoprenoid pathway. Similar efforts can be co-opted for the production of CoQ [63].

Conversely, in the eukaryotic platform, the mevalonate pathway begins with acetyl-CoA and ends with the similar production of IPP (Fig. 2). Midway through the pathway is the catalysis of HMG-CoA to mevalonate by HMGR, the target of statins. Unlike with statins, however, which seek to reduce HMGR activity, here aiming to increase its activity instead, so as to increase flux to the IPP pathway. Indeed, the lower K_m_ values of the downstream IPP pathway enzymes (farnesyl transferase and geranylgeranyl transferase) imply that the enzymatic reactions catalyzed (by enzymes including farnesyl and geranylgeranyl transferases) will reach saturation before that of HMG-CoA [7, 64]. This concept of exploiting HMGR for increased metabolic production is common; for example, a truncated HMGR lacking its inhibitory site can delay enzyme saturation [65, 66]. Regardless of the pathway source, downstream signaling leads to IPP and its isomer dimethylallyl diphosphate (DMAPP) (Fig. 2). IPP and DMAPP combine to form geranyl diphosphate (GPP), and this compound is sequentially lengthened by additional IPP moieties to form farnesyl diphosphate (FPP), geranylgeranyl diphosphate (GGPP), and the subsequent n-isoprene tail [61]. Depending on the host organism, components of the IPP pathway are also crucial branch points for several important compounds, which makes optimization of the isoprenoid pathway a lucrative endeavor (and one that has been done extensively in *S. cerevisiae* [59]). GPP can branch off and undergo reactions that lead to the formation of monoterpenoids; FPP, likewise, can form steroids and cholesterol; and GGPP can form carotenoids and retinoids before decaprenyl diphosphate [1]. Studies suggest that inhibiting these various branch points could direct metabolic flux from GPP towards decaprenyl diphosphate, as seen in FPP yields through the downregulation of squalene synthase [67].

CoQ_10_ production rates are thought to be limited by the availability of IPP, since the quinone head is produced from the relatively abundant chorismate or tyrosine [68, 69]. However, the tail length of CoQ, which contains varying numbers of IPP units, may also be rate-limiting. Although CoQ can be produced by multiple microbial platforms, each microbe synthesizes CoQ with a characteristic number of the IPP units. For example, *S. cerevisiae* and *E. coli* produce CoQ_6_ and CoQ_8_, respectively, whereas *S. pombe* and humans naturally produce CoQ_10_ [60]. Evidence shows that polyprenyl diphosphate synthase is the key determinant of IPP chain length, as this enzyme catalyzes polyisoprenoid tail extension [70]. In comparison, the polyprenyl diphosphate:4-HB transferase (UbiA/Coq2), which joins the tail and the quinone head, is promiscuous in terms of its isoprenoid chain length choice [71]. Therefore, any attempts to utilize a heterologous, non-native host to produce CoQ_10_ would need to optimize or replace the polyprenyl diphosphate synthase to achieve the appropriate tail length (10 isoprene subunits). Many groups have in fact approached this problem by introducing the decaprenyl diphosphate synthase (DPS) gene [72–74].

#### Rate-limiting steps in biosynthesis of the aromatic quinone group

Another likely avenue to increase metabolic flux is through the optimization of the aromatic quinone core. The precursor that contributes to the head group is 4-hydroxybenzoic acid (PHB or pHBA), which, in prokaryotes [60], forms from the condensation of phosphoenolpyruvate (PEP) and erythrose-4-phosphate, past shikimate, to chorismate and then PHB (Fig. 2) [68]. Chorismate is a branch point metabolite necessary in the formation of folate and aromatic amino acids (tyrosine and phenylalanine) [75]. Thus, it would be advantageous to increase the catalytic conversion of chorismate to PHB for both proper cell growth and metabolic flux [76].

Earlier work has also shown that CoQ production can be increased by the overexpression of chorismate pyruvate lyase (UbiC) in *E. coli* alongside the overexpression of several key catalytic enzymes that tend to limit CoQ production rates [77]. Similarly, an eightfold increase in CoQ_10_ was reported in the native producer *Sporidiobolus johnsonii* [78]. In other organisms, however, the source of PHB differs: mammals produce PHB from tyrosine, whereas yeast and plants use both chorismate and tyrosine (yeast) or a β-oxidation-like mechanism using p-hydroxycinnamic acid (plants) [60, 61]. In these cases, the exogenous addition of PHB can increase CoQ_10_ production; albeit, production rates are still reliant on the supply of IPP, which is rate-limiting [79, 80].

#### Rate-limiting steps in condensation of isoprenoid tail to the quinone group

In the final stages, polyprenyl-4-hydroxybenzoate transferase is required to combine the moieties to form the 4-hydroxy-3-polyprenylbenzoate precursor [60, 61, 81]. The isoprene group varies depending on the species, and the ring group undergoes a series of modifications (decarboxylation, hydroxylation and methylation) before the complete CoQ is synthesized. Flux is primarily determined by polyprenyl diphosphate transferase, and its overexpression in *E. coli* can generate a 3.4-fold increase in CoQ_10_ production [82]. Conversely, the overexpression of genes involved in ring modification leads to only a minor increase in CoQ_10_ content in *E. coli* and *S. pombe*, even if several genes are overexpressed together (in *S. pombe*) [83].

Overall, these findings suggest that the bottleneck in CoQ_10_ production still lies predominantly with IPP flux and is then limited by the quinone head formation and the required transfer steps [84].

### Host platforms employed for CoQ_10_ production

CoQ_10_ is only native to a few organisms [2, 81] and it remains unknown whether human metabolic reactions can cope with a shorter CoQ [85, 86].

Traditionally, most efforts have focused on native CoQ_10_ producers, and screening for mutant strains that show higher CoQ_10_ yields. However, there is great potential in exploiting heterologous hosts armed with extensive toolbox like *E. coli* and *S. cerevisiae* into platforms for CoQ_10_ production. Here, we explore the benefits and disadvantages of both native and non-native producers.

#### Native producers of CoQ_10_

Native producers have an advantage over heterologous hosts, as they do not produce any unwanted CoQ species (CoQ_8_ or CoQ_9_), which vary by chain length that and are specific to the host. The additional costs required to extract and separate CoQ_10_ from other shorter-tailed CoQ products may shift the balance in favor of using native producers of the enzyme. Indeed, these other, shorter products will compete for the biochemical flux and affect the yield of the desired CoQ_10_ [60].

Several native producers of CoQ_10_ have been identified or optimized as candidates for CoQ_10_ production, including *S. pombe*, *S. johnsonii*, *Rhodobacter sphaeroides* and *Agrobacterium tumefaciens* [78, 83, 87, 88]. Several other organisms, including *Pseudomonas*, *Paracoccus* bacteria, *Candida* and *Saitoella* yeasts also produce CoQ_10_ natively but have not been sufficiently characterized as producing hosts, and many require the inclusion of expensive constituents in the growth media for proper function. Here, we will explore four of the most feasible native hosts for CoQ_10_ production: (1) *S. pombe*, (2) *S. johnsonii*, (3) *R. sphaeroides* and (4) *A. tumefaciens*.

*Native producer: Schizosaccharomyces pombe*

*Schizosaccharomyces pombe* (fission yeast) is a well-studied model organism with similar molecular pathway makeup and genetic mechanisms as those in humans [89, 90]. However, little effort has been made to develop *S. pombe* into a suitable framework for high-value compound production [91], and so efforts to increase CoQ_10_ in *S. pombe* have thus far been limited. In one study, genes encoding enzymes directly involved in CoQ_10_ biosynthesis (*dps1* ^+^–*dlp1* ^+^, *ppt1* ^+^, and *coq3* ^+^–*coq9* ^+^) and HMGR [83] were overexpressed. However, only overexpression of HMGR—and not the CoQ_10_ biosynthesis genes—led to a prominent 2.7-fold increase in CoQ_10_ yield (Table 1). It was posited that the lack of effect from the biosynthetic genes was because these enzymes are not rate-limiting.

**Table 1:** Comparison of yield and methodologies employed in the native producers of coenzyme Q10

Native hosts for coenzyme Q_10_ production			
Host	Yield (mg/g DCW)	Ref	Methodologies
*Schizosaccharomyces pombe*	1.35	[83, 162]	Overexpression of native HMGR gene
*Sporidiobolus johnsonii*	10	[78]	Addition of exogenous HBA
*Rhodobacter sphaeroides*	12.96	[87]	Overexpression of multiple MEP pathway genes coupled with fine-tune of quinone modification pathway genes
*Agrobacterium tumefaciens*	6.92	[83, 163]	Ectopic expression of DXS and DPS genes in optimized media

More success has been attained in the production of ricinoleic acid, a fatty acid from castor oil in *S. pombe* [92], and it may be possible to hijack this system to co-produce both CoQ_10_ and fatty acids, with CoQ_10_ participating as a lipid-soluble antioxidant to protect polyunsaturated fatty acids (PUFA) against oxidative damage during storage. A similar approach has been explored in *Yarrowia lipolytica*, an oleaginous yeast, even though *Y. lipolitica* is a non-native producer of CoQ_10_, and this approach is currently undergoing approval for production [93]. The approach capitalizes on the same IPP pathway to produce carotenoids, and it has been suggested that this may lead to a reduction in flux and the generation of alterative products that will include CoQ_10_. Indeed, high CoQ_10_ selection based on mutant strains of *Protomonas extorquens* and *R. sphaeroides* are correlated with low carotenoid production [94].

*Native producer: Sporidiobolus johnsonii*

*Sporidiobolus johnsonii* was recently discovered as a natural producer of CoQ_10_ at 0.8–3.3 mg/g dry cell weight (DCW) (Table 1), which, in an unmodified strain, suggests a great potential as compared with the current top native (*A. tumefaciens*; 6.92–9.6 mg/g DCW) and heterologous (*E. coli*; 2.4 mg/g DCW; see below) producers [78, 95]. Efforts to use *S. johnsonii* as a production host at an industrial level have achieved 10 mg/g DCW; albeit, this yield involved exogenous PHB in the media [78]. Other mutagenesis attempts led to a mutant UF16 strain with 7.4 mg/g DCW [96].

*Native producer: Rhodobacter sphaeroides*

*Rhodobacter sphaeroides* is a photosynthetic bacterium [97] initially selected by screening mutant strains based on color change, which indicated a reduction in carotenoid production, and thus, by correlation, an increase in CoQ_10_ [94]. Promoter-based balancing of metabolic flux increased the production to 7.16–8.7 mg/g DCW [60, 98], and a recent study reported production as high as 12.96 mg/g DCW [87] (Table 1). However, other efforts to increase MEP pathway flux did not translate well into increased CoQ_10_ production, probably due to an accumulation of toxic intermediates [99]. *R. sphaeroides*, however, is reported to have limited growth rates, even when grown in optimal fermentation conditions [84]. This, coupled with other difficulties (such as requiring anaerobic and light conditions to produce higher CoQ_10_ titers) makes *R. sphaeroides* a less ideal host choice [100, 101].

*Native producer: Agrobacterium tumefaciens*

*Agrobacterium tumefaciens* is a Gram-negative bacterium that is widely used as a transmission vector tool for plant genetic modification [102]. Besides *R. sphaeroides*, it is one of the top producers of CoQ_10_ at 6.92–9.6 mg/g DCW [61, 83] (Table 1). Initial attempts to increase its production yield involved selecting cells based on their growth on inhibitory precursor analogues [103]. Later efforts involved targeting the overexpression of IPP pathway genes, especially *DXS* [60]. *A. tumefaciens*, however, produces unwanted exopolysaccharides, which increases the viscosity of the sample and affects CoQ extraction [88, 104].

*Issues with native hosts*

Native producers initially have higher CoQ_10_ yields as compared with non-native producers. However, few, if any, of the biosynthetic pathways leading to CoQ_10_ production have been optimized in these organisms, and the toolbox of promoters and genetic modules needed for effective tuning of native producers is lacking [84, 98]. Neither *A. tumefaciens* nor *R. sphaeroides* produce sufficient quantities of CoQ_10_ to meet current market demands, and this has led to higher prices of CoQ_10_ [38]. Furthermore, rather than optimizing the hosts, recent efforts in the field have been to develop toolkit pieces, such as promoter-regulated vectors [98, 99], or to determine ways to select for particular strains after mutagenesis [105]; only a few studies have attempted to harness metabolic engineering (to increase gene expression) or protein engineering [83, 87]. Other efforts garnered toward a more immediate solution have had to rely on the addition of precursors to increase yield, and this comes at a higher cost and therefore remains less feasible [106, 107].

#### Heterologous hosts

One method to circumvent the shortfalls seen with native producers is to use a heterologous platform that hosts high pliability towards genetic manipulation [108]. Heterologous systems are often avoided because of the production of unwanted CoQ species, the lengths of which are influenced by the chain length of the host organism and the nature of the heterologous polyprenyl diphosphate synthase; this is particularly complicated, as the synthases may function as either homo or hetero-dimers [109]. However, organisms that possess a large toolkit for host engineering are desirable, and their use holds promise to overcome some of the limitations seen with native hosts, assuming that these species can be appropriately optimized to produce CoQ with the correct chain length. In light of this, here, we explore two options—*E. coli* for prokaryotes and *S. cerevisiae* for eukaryotes [108]—as well as the utility of plants as heterologous hosts.

*Heterologous host: Escherichia coli*

The success in engineering *E. coli* to produce human insulin paved the way for a new frontier in metabolic engineering [110]. *E. coli* grows fast and is cheap to culture, and the large range of molecular tools, coupled with an extensive knowledge of its genetic, cellular and metabolic profiles, makes it a widely used production platform. Indeed, most compounds produced by metabolic engineering of *E. coli* command a good chance of success [108, 111, 112]. Hence, it is not surprising that strategies developed and optimized for the metabolite production in *E. coli* can be exploited for the production of CoQ_10_. However, *E. coli* natively produces CoQ_8_ not CoQ_10_ [77], and efforts to produce CoQ_10_ involved the addition of *DPS* from a native producer (*A. tumefaciens* or *G. suboxydans*) [113, 114]. Yet, despite producing CoQ_10_, the bacteria also produced CoQ products of variable tail lengths (CoQ_8_ and CoQ_9_) [115]. This was solved by knocking out the octaprenyl diphosphate synthase (IspB), which led to a minimal production of the other CoQ variants [116]. Other efforts used a *DPS* of greater stringency, and found that DPS from *R. sphaeroides* was more discerning in producing CoQ_10_ than DPS from *A. tumefaciens* [115].

Methods to improve the titer of CoQ_10_ in *E. coli* sought to increase the flux from the MEP pathway toward IPP [94, 116], while others reconstructed the complete mevalonate pathway to divert flux without encountering interference from negative regulators, such as HMGR by FPP in its native context [117, 118]. Although this reconstruction successfully increased CoQ_10_ yield, there was a metabolic bottleneck at the top end of the pathway involving mevalonate conversion (Fig. 2). When the lower part of the pathway was ectopically expressed, a twofold increase in yield was observed; yet, expression of the entire pathway led to only a 1.5-fold increase.

Several metabolomic studies in *E. coli* have investigated the rate-limiting steps in CoQ_10_ production [68, 119] by adding in the precursors exogenously to decouple the pathway away from cellular flux production. Not surprisingly, both the isoprenoid tail and aromatic quinone head are rate-limiting in *E. coli* [68, 120–122]. Yet, when these two precursors are no longer limiting, the downstream genes involved in ring modification (*ubiB*, *ubiH* and *ubiG*) becomes limiting [68]. In an effort to increase flux to the quinone precursor PHB, another study overexpressed chorismate pathway genes, including the gene encoding for 3-deoxy-d-arabinoheptulosonate 7-phosphate synthase, which initiates the first step in combining PEP with d-erythrose 4-phosphate [122] (Fig. 2). However, despite these efforts, CoQ_10_ levels in *E. coli* (0.45–3.63 mg/g DCW) still fall short of the levels produced by native producers (*R. sphaeroides* and *A. tumefaciens*) [99] (Table 2).

**Table 2:** Comparison of yield, benefits and limitations in the heterologous producers of coenzyme Q_10_

Host	Bacteria (*E. coli*)	Yeast (*S. cerevisiae*)	Plants
Yield	0.45–3.63 mg/g DCW	12.3 µg/g DCW	Not used
Suitability for human consumption	No	Generally recognized as safe (GRAS)	Yes
			Produced CoQ10 proposed to serve as direct dietary supplement
Fast growth	Yes	Yes	No
			Dependency on harvesting time
Extensive knowledge and tool-kit available for genetic, metabolic, protein engineering	Yes	Yes	No
Cultivation/culture density	High	High	Low
			Require large plot of arable land
Mixed chain length products produced and increase cost of purification of CoQ_10_	Yes	Yes	Yes
Inability to sidestep metabolic bottlenecks to induce high production level	Yes	Yes	Yes

*Heterologous host: Saccharomyces cerevisiae*

Another popular host in metabolic engineering efforts is the budding yeast, *Saccharomyces cerevisiae*. As a model organism, the genome of *S. cerevisiae* has been extensively studied and modified, and there are many already optimized tools for efficient gene expression and genetic building blocks for promoters and other regulatory elements [123, 124]. *S. cerevisiae* has a fast growth cycle of about 90 min, and has a high cultivable density as compared with bacteria. The budding yeast can also perform homologous recombination and compartmentalize subcellular processes, making it an excellent host for metabolic engineering purposes. It is also a ‘Generally Recognized As Safe’ (GRAS) organism (United States Food and Drug Administration) (Table 2), and this reduces any potential complications that could arise from its use in the production of a health supplement or a nutritional product [56]. Most importantly, the IPP pathway has been extensively optimized in *S. cerevisiae* [59].

Unfortunately, similar to *E. coli*, *S. cerevisiae* natively produces CoQ_6_ not CoQ_10_ [1]. Early attempts to delete the *COQ1* gene in *S. cerevisiae* and replace it with *DPS* from *G. suboxydans* under the *COQ1* promoter reportedly yielded 12.3 µg/g DCW [85]. However, DPS tends to require a heterodimer formation for proper function and, when expressed, may instead form dimers with native polyprenyl diphosphate synthases to produce products of differing lengths [125] (Table 2). An alternative approach would be to examine the functionality of the DPS enzyme by fine-tuning is length-determining function. This would be advantageous on several levels, given that the DPS reaction is a limiting step in CoQ_10_ production. Indeed, polyprenyl diphosphate synthase belongs to the protein family of prenyl-synthases, many of which are involved in generating the polyisoprenoid chain components of commercially interesting compounds like alkaloids and monoterpenes [7, 68]. If successful, this will conceptually sidestep the aforementioned problem of homodimerization of overexpressed heterologous DPS. We thus propose that an understanding of the mechanism by which polyprenyl diphosphate synthase determines chain length may allow for the production of CoQ_10_ in *S. cerevisiae* without generating off-target products.

*Heterologous host: plants*

Another suggested strategy for CoQ_10_ production is the use of plant hosts for the ease of CoQ_10_ supplementation into the diet [38]. Such efforts are currently underway, in conjunction with other nutritional supplements, such as vitamin A (beta-carotene) in ‘golden rice’ (*Oryza sativa*), which can be likewise co-opted in the context of CoQ_10_ given that carotenoid production also employs the IPP pathway [38, 126]. However, the political hassle associated with the commercialization of ‘golden rice’ or other genetically modified foods is expected to be a counter-rationale for the biosynthesis of CoQ_10_ in plant hosts [127–130]. Furthermore, because CoQ_10_ is also prescribed for deficiency-associated diseases and as an ingredient in various cosmetics, it must be properly extracted. Plant production hosts also have further technical obstacles, such as difficulties in engineering and manipulating the plant host; the need for large plots of expensive, arable land; a dependency on harvesting time; and the risk of unpredictable climate conditions in sync with market demand. It is for these various reasons that plant hosts are not deemed economically viable for CoQ_10_ production. These challenges, along with the comparatively less effort in the scientific community to exploit plant hosts, has meant that microbial hosts are a better choice for CoQ_10_ production [131].

### Potential future engineering approaches for CoQ_10_ production

There have been frequent attempts to engineer key enzymes within the CoQ_10_ pathway to increase the yield, including attempts to regulate IPP chain length. Recent interest in synthetic biology—which involves the fine-tuning of biosynthetic processes by controlling the genome and global organellar organization—promises to further revolutionize traditional bioengineering approaches. Several of the newly innovated methodologies will be discussed in the context of improving CoQ_10_ biosynthesis in the following sections.

#### Decaprenyl diphosphate synthase

In essence, there are two ways to induce a non-native heterologous host to make CoQ_10_: (1) Engineer the polyprenyl diphosphate synthase—which is solely responsible for chain length—to assume the function of DPS, or (2) introduce a DPS into the host and delete the native polyprenyl diphosphate synthase. The latter is based on earlier reports, where CoQ of differing tail lengths have been produced by heterologous hosts [2, 15, 71, 74]. Specifically, the introduction of *ddsA* and *sdsA* into *E. coli* from *G. suboxydans* and *Rhodobacter capsulatus*, respectively, can result in the formation of CoQ_10_ (and also CoQ_9_) [74, 113, 132]. PHB-polyprenyl diphosphate transferase (COQ2) lacks the specificity of polyprenyl diphosphate synthase, as it is able to transfer isoprenoid tails of varying length; e.g., *E coli* UbiA can utilize isoprenoid chains of 5–10 residues in length. Based on this promiscuity, the PHB:polyprenyl diphosphate transferase is expected not to be a limiting factor in engineering a non-native host for the production of CoQ_10_.

However, engineering a heterologous host via the introduction of exogenous DPS suffers from challenges that cannot, as yet, be explained. Even with the efforts of removing endogenous CoQ production by deleting the native polyprenyl diphosphate synthase gene, there remains a lack of stringency in these reactions. For example, when the DPS gene from *G. suboxydans* is expressed in *E. coli*, deletion of the native IspB gene only reduces the production of CoQ_8_ and CoQ_9_; even though it still predominantly produces CoQ_10_ [74, 113]. A further complication to the engineering effort lies with the complex formation of polyprenyl diphosphate synthases, which function as homodimers (IspB in *E. coli*, Coq1 protein in *S. cerevisiae*, and DdsA in *G. suboxydans*) or heterotetramers (Dps1-Dlp1 in *S. pombe* and HsPDSS1-HsPDSS2 in humans) [133, 134]. For instance, when heterologously expressed in *E. coli*, *COQ1* from *S. cerevisiae* can replace IspB, an otherwise essential gene for the production of CoQ_6_ [70, 132]. However, when *COQ1* from *S. cerevisiae* is expressed in Dlp1-deficient *S. pombe*, it rescues the *dlp1* deletion by forming a heterodimer with Dps1 to produce CoQ_10_ [131]. Similarly, Dps1 or Dlp1 in *S. pombe* can complex with defective IspB mutants to restore functionality in *E. coli* [135]. In such cases, heterologous expression of DPS creates artificial interactions with the host DPS, calling for caution in considering CoQ_10_ production through host chassis engineering.

#### Polyprenyl diphosphate synthase residue functionality

Polyprenyl diphosphate synthases catalyze the formation of the polyprenoid tail by adding IPP units to an allylic diphosphate base [136]. These enzymes are categorized depending on the final carbon chain length of the synthesized product: class I for C10–20, class II for C30–35, class III for C40–50, and class IV for even longer products [115]. Class IV synthases also catalyze some *cis*-configuration double bonds, whereas the other classes all catalyze *trans*-configuration bonds. Synthases from class II and III categories should be chosen when studying tail length determination because these classes reflect both the final carbon chain length product and possess a similar stereo configuration of double bonds to that of DPS, with an average homology of 30–50% between the polyprenyl diphosphate synthases and DPS enzymes [113, 133].

**Fig. 3:**
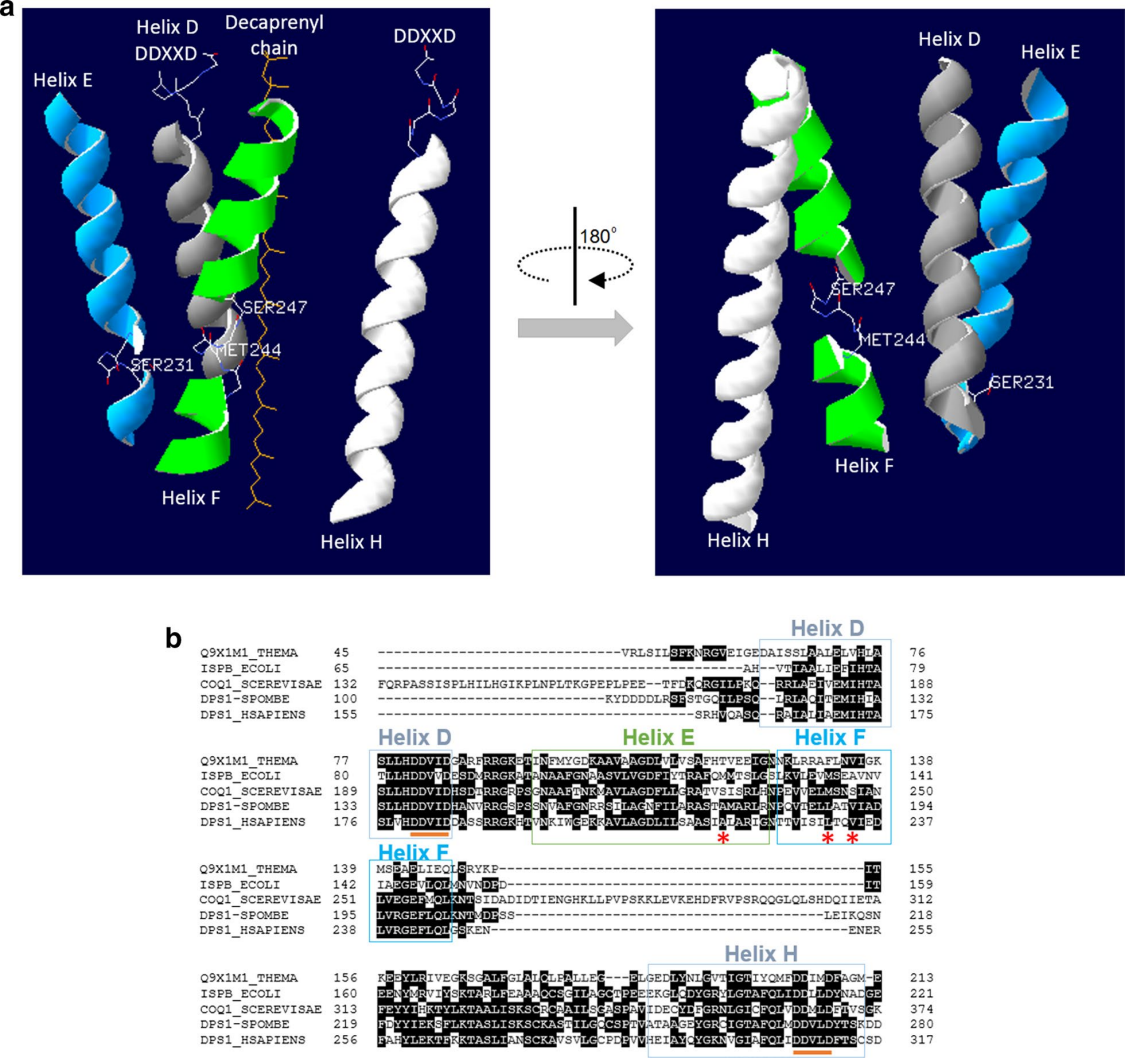
**a** Protein homology modeling of COQ1 (YBR003W) was performed using ModBase [159] and was viewed using Swiss PDB Viewer [160]. The template for modeling was based on the medium/long-chain length prenyl pyrophosphate synthase of *Arabidopsis thaliana* (3aq0A) with 42% sequence identity. Helix D and Helix H bind to the elongating isoprene chain and IPP, respectively, at the conserved DDXXD regions. Helix F contains Met-244 and Helix E contains Ser-231, which are thought to be the residues that regulate chain length elongation. The *right figure* represents the 180° view of that on the *left* and is superimposed with the structure of CoQ_10_. **b** Multiple sequence alignment of Q9X1M1_THEMA (*T. maritime* TM_1535), ISPB_ECOLI (*E. coli* IspB), COQ1_SCEREVISIAE (*S. cerevisiae* COQ1), DPS1_SPOMBE (*S. pombe* Dps1) and DPS1_HSAPIENS (Human PDSS1) using CLUSTAL W [161]. Helices D (*grey*), E (*green*), F (*blue*), and H (*white*) indicated in (**a**), are boxed in (**b**). *Orange underline marks* the DDXXD motif. *Red asterisks* indicate the positions of *S. cerevisiae* COQ1 Met-244, Ser-247 and Ser-231 residues. Met-244 corresponds to Leu-188 and Leu-231, and Ser-247 to Val-191 and Val-234 of *S. pombe* Dps1 and *H. sapiens* PDSS1, respectively. Labels of helices are marked with the *same colors* as those used for the helices in **a**

There are seven conserved regions within *trans*-type prenyltransferases, two of which (domain II and VI) possess a DDXXD motif [71, 137]. These motifs are located in two helices that face each other, and are the binding sites for FPP (Helix D) and IPP (Helix H) with the aid of Mg^2+^ in substrate binding [136, 137] (Fig. 3a). The fifth residue before the DDXXD motif in domain II determines tail chain length. In GGPP and FPP (*Thermoplasma*), this residue is Tyr-89, a large bulky residue; in OPP (*Thermotoga maritime*) and IspB (*E. coli*), it is Ala-76 and Ala-79, respectively. These amino acid differences are associated with an inverse relationship between residue size and chain length [136]. Indeed, when Ala-76 and Ala-79 are changed to Tyr, the product chain length decreases from C40 to C20 [136]. In another study, this same substitution (in *E. coli*) in the absence of wild-type IspB, produces a non-functional protein, but one that is still able to heterodimerize with the wild-type protein to produce CoQ_6_ [71].

Elongation of the polyprenyl chain takes place in a ‘tunnel’ between helices H and D, where A76 (in *T. maritime*) lies at one end near the DDXXD motif and Phe-132 (Met-135 in *E. coli*) lies at the other end; this Phe residue is thought to serve as a cap-like residue [136, 138]. Mutating Phe-132 to Ala can increase the chain length from C40 to C50, which suggests a method to increase chain length synthesis by polyprenyl diphosphate synthases. This was confirmed by others, who, using a *cis*-type prenyltransferase, found that substituting leucine for alanine increased the chain length from C55 to C70 [139]. In addition to Met-135 in *E. coli*, another residue, Met-123, compositely serves to limit the elongation of the IPP chain; hence, Met-123 and Met-135 are proposed to contribute to a ‘double-floor’ (‘floor’ is synonymous with ‘barrier’), as opposed to the ‘single-floor’ created by Phe-132 in *T. maritime* [138].

Efforts to engineer the polyprenyl diphosphate synthases in a host that is highly malleable to genetic and metabolic engineering, such as *S. cerevisiae*, may provide a prospective avenue to increase the yield of CoQ_10_. A sequence alignment of polyprenyl diphosphate synthases from various organisms shows high similarity at the amino acid level in the helices that constitute the chain elongation tunnel (Fig. 3b). Such high conservation means that functional studies conducted with *T. maritime* polyprenyl diphosphate synthases could serve as a reference to guide engineering efforts in other species, such as *COQ1* from *S. cerevisiae*.

#### Spatial metabolic organization with synthetic compartmentalization

Metabolic production of CoQ_10_ may also be increased by manipulating the spatial organization of the enzymes in the cell. This is particularly important when faced with potential off-target reactions or when the accumulation of products results in toxicity [140–142]; albeit, clinical studies indicate that toxicity from CoQ_10_ supplementation is not a huge concern [29].

Some of the more common ways to recruit the pathway into a localized complex involves the use of protein scaffolds or linkers to tether the pathway enzymes to proteins of interest [143–147]. This manipulation concentrates the substrate close to the enzyme, and may favor the forward metabolic flux, as an intermediary metabolite may be captured and shunted into the next step of the pathway. Conceptually, such spatial arrangement reduces the emergence of unwanted by-products, especially with more promiscuous enzymes.

When stoichiometric ratios of sequential reactions are of relevance, tethering also helps to modulate the ratio of enzyme to protein [56]. However, tethering may cause rigidity in the protein scaffold or direct enzyme fusions that could affect enzymatic function. However, these issues can be overcome with the use of a linker sequence, which provides increased flexibility to orientate the direction of the reaction and lower the risk of potential disruptions to enzyme folding.

**Fig. 4:**
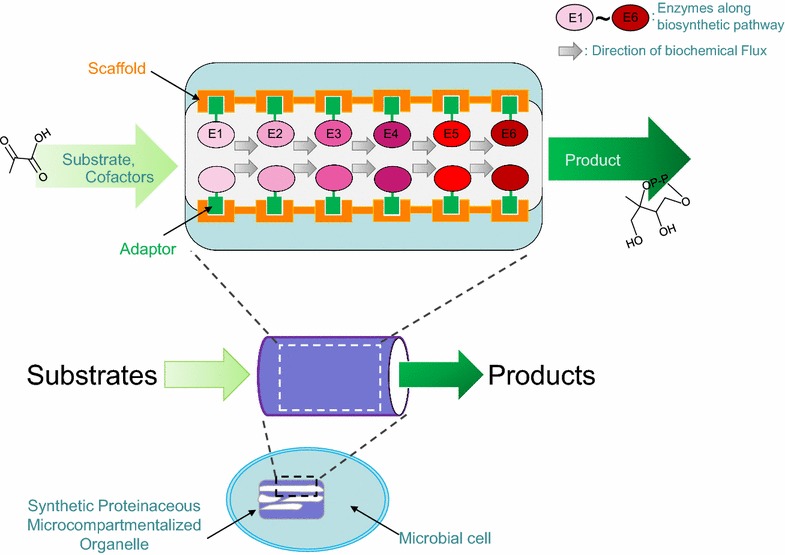
Spatial metabolic organization with synthetic compartmentalization. Diagrammatic representation of a synthetic proteinaceous or nanotube micro-compartmentalized organelle can be engineered in microbial cells [149–151]. The organelle consists of a scaffold on which the biosynthetic enzymes can be immobilized to direct the biochemical flux such that the substrate of an enzyme is the product of another juxtaposed enzyme. Toxic byproducts may conceptually be shunt into sub-compartments within the organelle and sequester therein to ensure optimal growth of the microbial host

In lieu of scaffold or linker systems, synthetic subcellular compartmentalization can also be used, whereby the enzyme complex is targeted to protein shells or organelles (Fig. 4). This would further reduce any unwanted side-effects or steric problems, which likely occur on protein scaffolding. The use of such synthetic compartmentalization may also sequester any toxic products produced by the reaction and preserve cell viability. One potential pathway for the ectopic induction of compartmentalization is through the use of bacterial microcompartments—proteinaceous organelles derived from prokaryotes [148–151]. These synthetic organelles possess selectively permeable surfaces comprising thousands of shell proteins and can sequester the enzymatic pathways by means of N-terminal targeting sequences to link the enzymes to the surface of the organelle. Carboxysomes are one example of a bacterial microcompartment that contains ribulose-1,5-bisphosphate carboxylase oxygenase (RuBisCO) for carbon-fixing activities [152–154]. In eukaryotes, protein-based compartments (which comprise ribonucleoprotein particles) known as ‘vaults’, can also be used; albeit, less is known about the structure and mode of formation of these compartments [155, 156]. Finally, it may be simpler to target the eukaryotic organelle pathways that already exist; for instance, one group sought to increase opioid production by altering the pathway of proteins to the endoplasmic reticulum (ER) by ER-tagging of the relevant enzymes. This modification increased the titer and specificity of the product of interest [157].

In cases where modifications are made to the pre-combined quinone head and isoprene tail, the enzymes required are already localized in the mitochondria in a membrane-bound complex (eukaryotes) or on the cell membrane (prokaryotes); although, there is, as yet, no evidence for a complex in prokaryotes [11, 158]. However, the other pathways involved in generating the precursor head and which lack bio-orthogonal chemistry are still candidates for spatial organization; for example, the mevalonate pathway, which leads to the IPP precursor, could be one option. Indeed, SH3 ligands and domains are used to link HMG-CoA synthase with HMGR to prevent the accumulation of HMG-CoA and reduce its associated cytotoxicity [143].

Chorismate could be another option. As mentioned earlier, chorismate is a branch point metabolite and thus its recruitment could be spatially separated so as to prevent its conversion into off-target aromatic amino acids. This segregation would be advantageous, as this pathway is essential and cannot be completely disrupted. If a plant platform were to be used, attention would have to be given to the alternate and possibly competing products of GPP, FPP and GGPP. In non-native hosts, CoQ products will present with a range of tail lengths because of the use of the promiscuously inserted decaprenyl diphosphate synthase and its interactions with host polyprenyl diphosphate transferase. These are some possible candidate biosynthesis modules that may benefit from the manipulation of spatial organization and can be optimized in future experiments.

## Conclusions

CoQ_10_ is a valuable and commercially important product that has yet to be produced to a level that can support market demands. This review gives an overview of the native and heterologous hosts reported thus far for the production of CoQ_10_. Currently *Rhodobacter sphaeroides* triumphed as the native host in producing 12.96 mg/g DCW of CoQ_10_. On the other hand, the most widely used workhorse for industrial production of valuable compounds—*E. coli*—only achieved 3.63 mg/g DCW as the most productive of the heterologous hosts by far. Thus, use of native hosts still remains as the best option for industrial scale production of CoQ_10_. However, with new tools and progress made in recent years with the advent of synthetic biology, CoQ_10_ production may stand a chance to be revolutionized. It will be exciting to expect future new technological breakthroughs in this field to take production to new levels either in native or heterologous producers.

## Abbreviations

CoQ: coenzyme Q
CoQ_10_: coenzyme Q with chain containing 10 isoprene subunits
VLDL: very low density lipoproteins
CNS: central nervous system
PDSS1: prenyl diphosphate synthase, subunit 1
HMG-CoA: 3-hydroxy-3-methylglutaryl-coenzyme A
SAM: s-adenosyl methionine
MEP: 2-C-methyl-d-erythritol 4-phosphate
G3P: glyceraldehyde 3-phosphate
DXP: 1-deoxy-d-xylulose 5-phosphate
DXS: 1-deoxy-d-xylulose-5-phosphate synthase
DXR: 1-deoxy-d-xylulose 5-phosphate reductoisomerase
IPP: isopentenyl diphosphate
HMGR: HMG-CoA reductase
DMAPP: dimethylallyl diphosphate
GPP: geranyl disphosphate
FPP: farnesyl diphosphate
GGPP: geranylgeranyl diphosphate
DPS: decaprenyl diphosphate synthase
PHB or pHBA: 4-hydroxybenzoic aid
PEP: phosphoenolpyruvate
DCW: dry cell weight
GRAS: generally recognized as safe
RuBisCO: ribulose-1,5-bisphosphate carboxylase/oxygenase
